# Expanding Chemical Probe Space: Quality Criteria for Covalent and Degrader Probes

**DOI:** 10.1021/acs.jmedchem.3c00550

**Published:** 2023-07-05

**Authors:** Ingo V. Hartung, Joachim Rudolph, Mary M. Mader, Monique P. C. Mulder, Paul Workman

**Affiliations:** † Medicinal Chemistry, Global Research & Development, Merck Healthcare KGaA, 64293 Darmstadt, Germany; ‡ Discovery Chemistry, Genentech, South San Francisco, California 94080, United States; § Molecular Innovation, Indiana Biosciences Research Institute, Indianapolis, Indiana 64202, United States; ∥ Department of Cell and Chemical Biology, Leiden University Medical Center, 2333 ZA Leiden, The Netherlands; ⊥ Centre for Cancer Drug Discovery, The Institute of Cancer Research, London, Sutton SM2 5NG, United Kingdom; # Chemical Probes Portal, https://www.chemicalprobes.org/

## Abstract

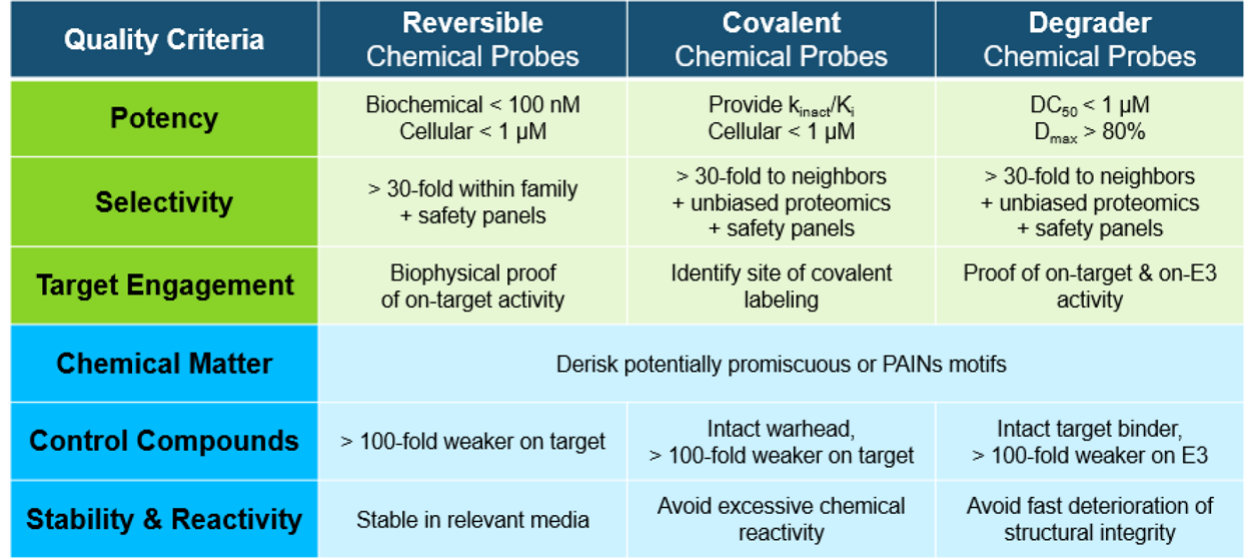

Within druggable target space, new small-molecule modalities, particularly covalent inhibitors and targeted degraders, have expanded the repertoire of medicinal chemists. Molecules with such modes of action have a large potential not only as drugs but also as chemical probes. Criteria have previously been established to describe the potency, selectivity, and properties of small-molecule probes that are qualified to enable the interrogation and validation of drug targets. These definitions have been tailored to reversibly acting modulators but fall short in their applicability to other modalities. While initial guidelines have been proposed, we delineate here a full set of criteria for the characterization of covalent, irreversible inhibitors as well as heterobifunctional degraders (“proteolysis-targeting chimeras”, or PROTACs) and molecular glue degraders. We propose modified potency and selectivity criteria compared to those for reversible inhibitors. We discuss their relevance and highlight examples of suitable probe and pathfinder compounds.

## Significance

- High-quality chemical probes are important tools which allow generation of robust and reproducible insights into the cellular function of proteins of interest.
- Covalently acting small molecules and small-molecule protein degraders extend druggable space beyond that fraction of the proteome which is targetable with reversibly acting ligands.
- When applied, the proposed set of quality criteria increases the likelihood that cell biology studies provide robust insights of high basic and translational relevance.

## 1 Introduction

Small molecules (“chemical probes”) designed to selectively modulate a protein of interest (PoI) play an important role in understanding protein function and validating new drug targets.^1^ Such chemical probes allow the translation of genetic and biological studies into meaningful experimental evaluation of therapeutic interventions. While some chemical probes are drugs, not all drugs have the characteristics needed to confidently validate a novel biological target. Thus, developing probes is, in itself, a significant discovery effort.

Low reproducibility and robustness of target validation studies was recognized more than a decade ago as a contributing factor for lower-than-desired productivity of drug discovery and development.^2^ The problem was in part attributed to low-quality tools for target validation studies, including insufficiently characterized small molecules, and has led to concerted efforts to raise awareness and make high-quality chemical probes accessible to the scientific community.^3^

**Figure 1:**
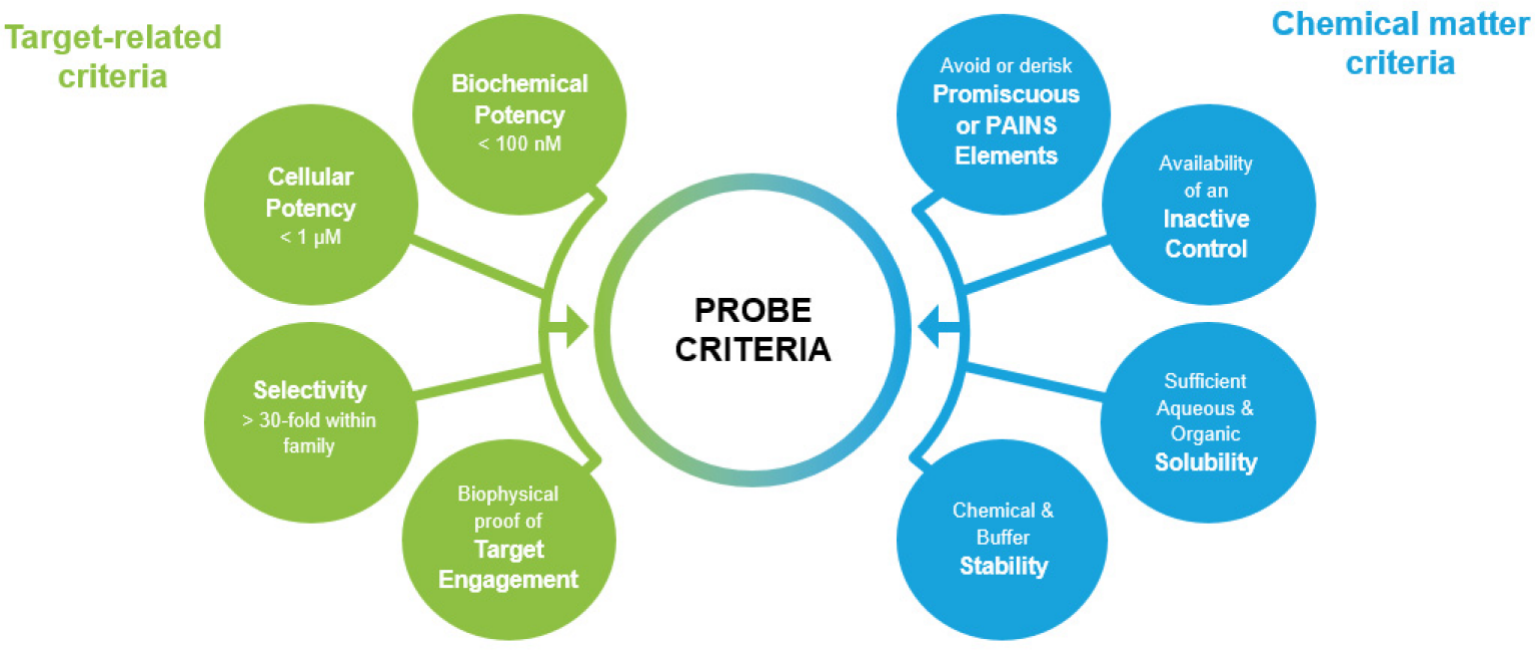
Quality criteria for reversibly acting small-molecule modulators of proteins. Adapted from ref (4), published under a Creative Commons License.

Applying quality criteria has by now been broadly accepted as best practice when selecting reversibly acting small-molecule modulators of enzymatic function and protein–protein interactions (PPIs) for cell biology studies. One broadly used set of criteria for such chemical probes was proposed by the Structural Genomics Consortium (SGC) and collaborators (Figure 1; adapted from ref (4)).^5−7^ It comprises a set of biology-focused criteria (biochemical and cellular potency, target selectivity, and proof of target engagement) and a set of chemical-matter-related criteria (absence of moieties causing overt promiscuity or assay interference, sufficient solubility and stability to be usable under typical assay conditions, and availability of a chemically similar but inactive negative control molecule).

For intracellular targets, small molecules continue to be the drug modality of choice due to their ability to cross cell membranes and reach all intracellular compartments. However, only a fraction of the human proteome can be interrogated with reversibly acting small molecules. The quest to identify modulators for a larger fraction of the human proteome has led to the exploration and use of additional drug modalities beyond reversibly acting small molecules.

Research on covalently acting small molecules, both as drugs for clinical use and as tools to study protein functions, has surged in the past decade.^8^ Renewed interest in using reactive groups (“warheads”) in small-molecule drug discovery initially focused on enzymes with nucleophilic amino acid side chains in their active sites, leading to the approval of the protease inhibitor boceprevir and the proteasome inhibitors bortezomib and carfilzomib (inspired by the natural product epoxomicin). A watershed moment for covalent drug discovery was the approval of the cysteine-targeting BTK inhibitor ibrutinib, a compound with significant clinical impact.^9^ Progress in using MS-based proteomics technologies for activity-based protein profiling (ABPP) approaches in a cellular context^10^ and, more recently, high-throughput crystallography with electrophilic fragments^11^ has led to a wave of small-molecule covalent binders for so far undrugged targets.

In parallel, small molecules have been employed to induce proximity between a PoI and proteins involved in the ubiquitin proteasome system (mostly E3 ligases) that lead to the degradation (“targeted protein degradation”, or TPD) of the former. The two main classes of such molecular degraders, bifunctional proteolysis-inducing chimeras (PROTACs) and molecular glue degraders (e.g., the clinically used immuno-modulatory drug (IMiD) and sulfonamide classes), have gained significant traction in drug discovery.^12,13^ Such molecular protein degraders recapitulate genetic knock-down phenotypes and therefore have the potential to become exceptionally valuable tools to interrogate protein function.^14,15^ Both new modalities provide a quantifiable biomarker for target engagement *in vivo* by measuring either the fraction of covalently labeled protein or the level of protein degradation.

As with reversible small-molecule probes, the quality of covalent molecular probes and molecular degrader probes will define the value generated by cell biology studies with such tools, and scientific rigor is required in selecting appropriate molecules. However, the scientific community has not yet developed a joint understanding about quality criteria for covalent and degrader probes, although first sets of guidelines have been proposed.^15−18^

Starting from the well-accepted criteria for reversible molecular probes, we discuss important quality aspects and propose criteria for covalent and degrader probes. The present authors make use of several decades of experience in discovering and using chemical probes in both academic and industrial settings. We focus on aspects that are of highest importance for the users of such probes: 1) What information is necessary to use a covalent or degrader probe with confidence? 2) What needs to be considered when drawing conclusions from cellular studies with such probes? 3) What are red flags that indicate a lack of probe fitness for biological research and target validation purposes?

## 2 Quality Criteria for Covalent Probes

**Figure 2:**
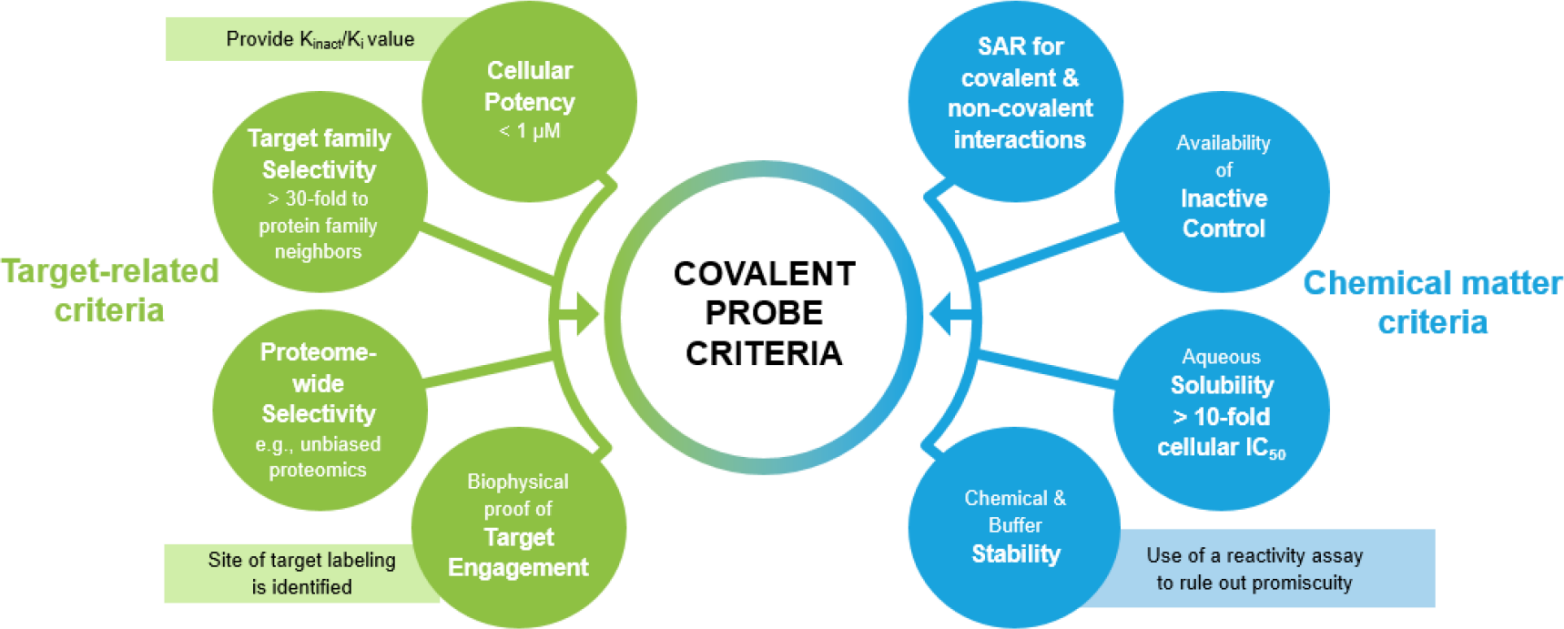
Proposed quality criteria for covalently acting small-molecule probes.

In 2004, Christopher Lipinski—famous for proposing the Rule of 5 for oral drugs—recommended that covalent chemistry should be avoided in tool compounds for target validation work.^19^ Today it is broadly appreciated that covalent conjugation represents an important expansion of the repertoire of drug hunters to target poorly ligandable proteins. However, significant efforts need to be invested into characterizing and validating covalent molecules as a prerequisite for conclusive use in biomedical research and target validation studies.^16^ In addition, covalent drug discovery also impacted highly druggable protein families, such as kinases.^20^ We propose a set of quality criteria for covalently acting small-molecule probes in Figure 2. We will focus our discussion on covalent targeting of cysteine residues, as this is currently of highest relevance for the users of chemical probes, although other nucleophilic amino acids can be targeted.

### 2.1 Criteria for Assessing Potency of Covalent Probes

When working with irreversible covalent probes, it is important to consider that target inhibition is time-dependent and therefore IC_50_ values, while frequently used, are a suboptimal descriptor of potency.^21^ Best practice is to use *k*_inact_ (the rate of inactivation) over *K*_i_ (the affinity for the target) values instead.^22^ Fully optimized covalent drugs can achieve *k*_inact_/*K*_i_ values greater than 1 × 10^5^ M^–1^ s^–1^, but no cutoff for a desirable potency range has been proposed yet, as these values are highly dependent on the PoI. Efforts to target KRasG12C and EGFR are especially instructive, and reported *k*_inact_/*K*_i_ values for representative inhibitors are compiled in Table 1.

**Table 1:** *k*_inact_/*K*_i_ Relative to Active Concentration in Cellular Assays for Representative Covalent Inhibitors of KRasG12C and EGFR

Compound	Target	*k*_inact_/*K*_i_ [s^–1^ M^–1^]	Active concentration in cellular assays [nM]	Ref
Shokat Lead Cmpd 12	KRasG12C	0.33	≥10,000	(23)
ARS-853	KRasG12C	250	2,500–10,000	(23)
ARS-1620	KRasG12C	1,100	250–1,000	(23)
MRTX849 (Adagrasib)	KRasG12C	35,000	5–68	(27)
AMG510 (Sotorasib)	KRasG12C	9,900	5–14	(28)
RMC6291	KRasG12C	289,000	50	(29)
GDC-6036	KRasG12C	27,000	0.2	(30)
JDQ443	KRasG12C	141,000	20	(26)
BI-0474	KRasG12C	15,220	7–26	(31)
Afatinib	wt EGFR	6.3	11.5	(22)
Afatinib	L858R/T790M EGFR	15	7.3	(22)
Osimertinib	wt EGFR	28,000	480^a^	(33)
Osimertinib	L858R EGFR	570,000	15–17^a^	(33)

a From ref (32).

The combination of high chemical reactivity with low reversible affinity is undesirable for a covalent probe due to the increased risk of polypharmacology. Importantly, *k*_inact_ is not equivalent to chemical reactivity as it is governed by the structural environment surrounding the conjugated amino acid, its resulting nucleophilicity, and the geometric orientation of the presented warhead.^23^ As measurement of *k*_inact_/*K*_i_ values can be labor-intensive (or in certain cases technically impossible), IC_50_ values (or target engagement TE_50_ values) are often reported for covalent leads and used to generate structure–activity relationships (SARs). Carefully designed biochemical assays used in determining IC_50_ values can be well-suited as surrogates for *k*_inact_/*K*_i_ measurements.^24^

We recommend, when relying on IC_50_ values, to report values for different time points for selected key compounds and to correlate reported IC_50_ values to data from cellular functional assays. Measuring the rate of protein resynthesis in relevant cell lines can be useful to predict the likely functional impact of covalent inhibition of the target. Whenever relying on cellular assays for optimization, the potential contribution by off-targets needs to be kept in mind, especially when relying on “down-assays” (reduction in assay signal upon compound treatment), as compounds impacting fitness of the cell line may generate strong assay signals.^25^ We therefore recommend cellular assays that report biochemical effects that are as proximal as possible to the target protein’s function.

**Figure 3:**
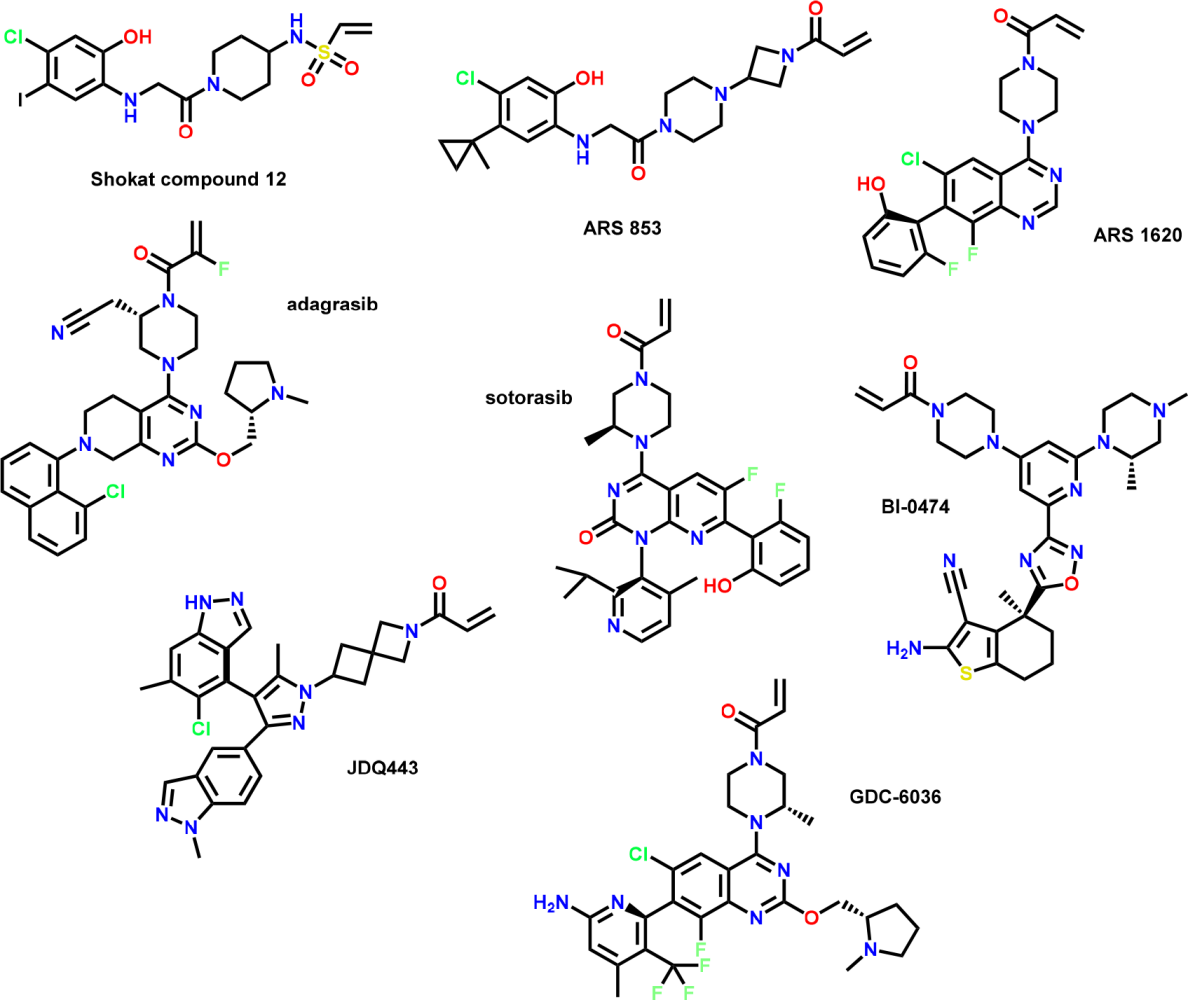
Covalent KRasG12C inhibitors represented in Table 1.

For example, it was shown that for covalent inhibitors of KRasG12C from one chemical series, the correlation of *k*_inact_/*K*_i_ values with cellular potency allowed the primary use of cellular potency values to drive optimization.^26^ Of note, structurally diverse KRasG12C inhibitors with *k*_inact_/*K*_i_ > 1000 showed cellular functional activities at concentrations below 1 μM (Table 1, chemical structures shown in Figure 3). In addition to assessing on-target reactivity, assays measuring intrinsic (chemical) reactivity are of value to assess the quality of covalent chemical probes (see below).

Because of the difficulty of fully capturing the potency of covalent probes in biochemical assays, target-dependent activity in cellular assays has become an even more important quality criterion. We recommend, aligned with the established best practice for reversible chemical probes, that high-quality covalent probes should show cellular target engagement at concentrations below 1 μM. Where direct cellular target engagement is difficult to assess, proximal functional biomarkers may be used instead. Achieving cellular target engagement at concentrations below 1 μM allows the use of such a probe at such low concentrations for target biology studies. The use of high μM concentrations of covalently acting probes in cellular assays should be avoided, as this increases the risk of coincidental engagement of off-targets. Due to the importance of kinetics, incubation and read-out times for cellular assays may need to be adapted to match the characteristics of the specific probe. For example, information on the time needed to reach maximum target occupancy can help to select (pre)incubation periods before administering a cellular stimulus or assessing a functional read-out. Wash-out experiments can provide evidence that the covalent mode-of-action is driving observed cellular phenotypes.^34^

### 2.2 Criteria for Assessing Covalent Probe Selectivity

It is crucial for a high-quality covalent probe to have one primary site of covalent interaction, that this site has been mapped to the PoI, and preferably that it has been shown that point-mutating this amino acid, if tolerated, prevents labeling of the PoI (or blocks functional cellular effects).^35^ Knowing the site of labeling enables interrogation of homologous or similar sequences in related proteins and rationally guided assessment of selectivity. We propose a selectivity factor of 30-fold in favor of the intended target of the probe compared to that of other family members or identified off-targets under comparable assay conditions.

In addition, selectivity for covalent probes needs to be assessed in an unbiased way, for example by unbiased MS-proteomics studies identifying labeled proteins proteome-wide.^36^ Pull-down experiments (with biotin-labeled or clickable derivatives) are another powerful approach to identify off-targets in an unbiased way. As for other probes, a recommended concentration range to be used in cellular studies should be provided by authors, and data need to be generated validating that potential off-targets are not engaged in this concentration range. Reactivity assays can be used to filter out potentially promiscuous molecules (see below).

### 2.3 Chemical Matter Criteria for Covalent Probes

Ideally, the on-target activity of the covalent probe is not dominated by the reactive warhead, but the rest of the molecule provides a measurable reversible affinity for the intended target. Seeing SARs over 1–2 log units of activity resulting from core, substitution, and warhead changes is an important quality criterion for covalent probe molecules.

Providing a control compound with significantly reduced on-target activity can increase the value of a covalent probe. For covalent probes, two types of contributions are at play: the rate of inactivation and the affinity of the ligand; thus, both aspects must be considered. From our experience, it is not sufficient to provide a matched analog devoid of the reactive group (e.g., acetyl instead of α-chloro-acetate, or saturation of a Michael acceptor motif), although such an analog may help to quantify the covalent contribution to probe activity. We believe that the more valuable control compound is a matched analog that retains the unchanged reactive group but with modifications in other parts of the molecule leading to >100-fold reduction in potency against the PoI. Making use of small substituents leading to clashes with the binding site or inverting stereocenters while keeping the warhead intact are broadly employed strategies to identify such control compounds. Stereoisomeric mixtures may already be used at the screening stage of probe discovery to directly identify matched control compounds.^37,38^ Unchanged cellular effects with such a control molecule indicate that off-targets may be driving the phenotype, while reduced cellular effects would be consistent with loss of on-target activity.

It is difficult to define a general cutoff for overall reactivity of the warhead beyond what is dictated by practical reasons: sufficient stability in water, buffer, and optionally plasma to allow for the performance of functional studies. We recommend that stability data are reported for key assay buffers with a recommended threshold of >80% stability for the used incubation time in reported assays. If the stability is much lower, then observed cellular phenotypes cannot be attributed with certainty to the probe molecule.

The reaction rate with cysteine or glutathione (GSH) can provide a measure of intrinsic reactivity. Corresponding assays can provide an indirect way to assess probe specificity and help to filter out compounds that will likely be promiscuous. For example, at Genentech, a cysteine reactivity assay is extensively used in covalent drug discovery programs, and a *t*_1/2_ < 5 min typically signifies undesirable compounds. Due to the experimental simplicity of such an assay, its use in covalent drug discovery campaigns is highly recommended and has become standard practice in industrial settings. As assay conditions can have a major impact on reaction rates, absolute numbers from different publications should typically not be compared. As mentioned above, intrinsic chemical reactivity must not be confused with the rate of inactivation. Covalently acting molecules can have very high rates of inactivation while showing only moderate intrinsic warhead reactivity. For example, the KRasG12C inhibitor sotorasib is reported to have an extremely high *k*_inact_ of 0.85 s^–1^, while the compound shows low intrinsic chemical reactivity, with a half-life in the presence of 5 mM GSH of 200 min.^39^

The intrinsic reactivity of the warhead will typically be tuned during the optimization of the compound. Others have reviewed warhead reactivity^20,40^ and have observed that warheads such as chloroacetamides and acrylamides derived from anilines are typically too reactive to be found in selective probes or drugs, while α-halopropionamides and acrylamides derived from alkylamines have the potential to be in the desired range.

### 2.4 Examples of High-Quality Covalent Probes and Covalent Pathfinder Probes

**Figure 4:**
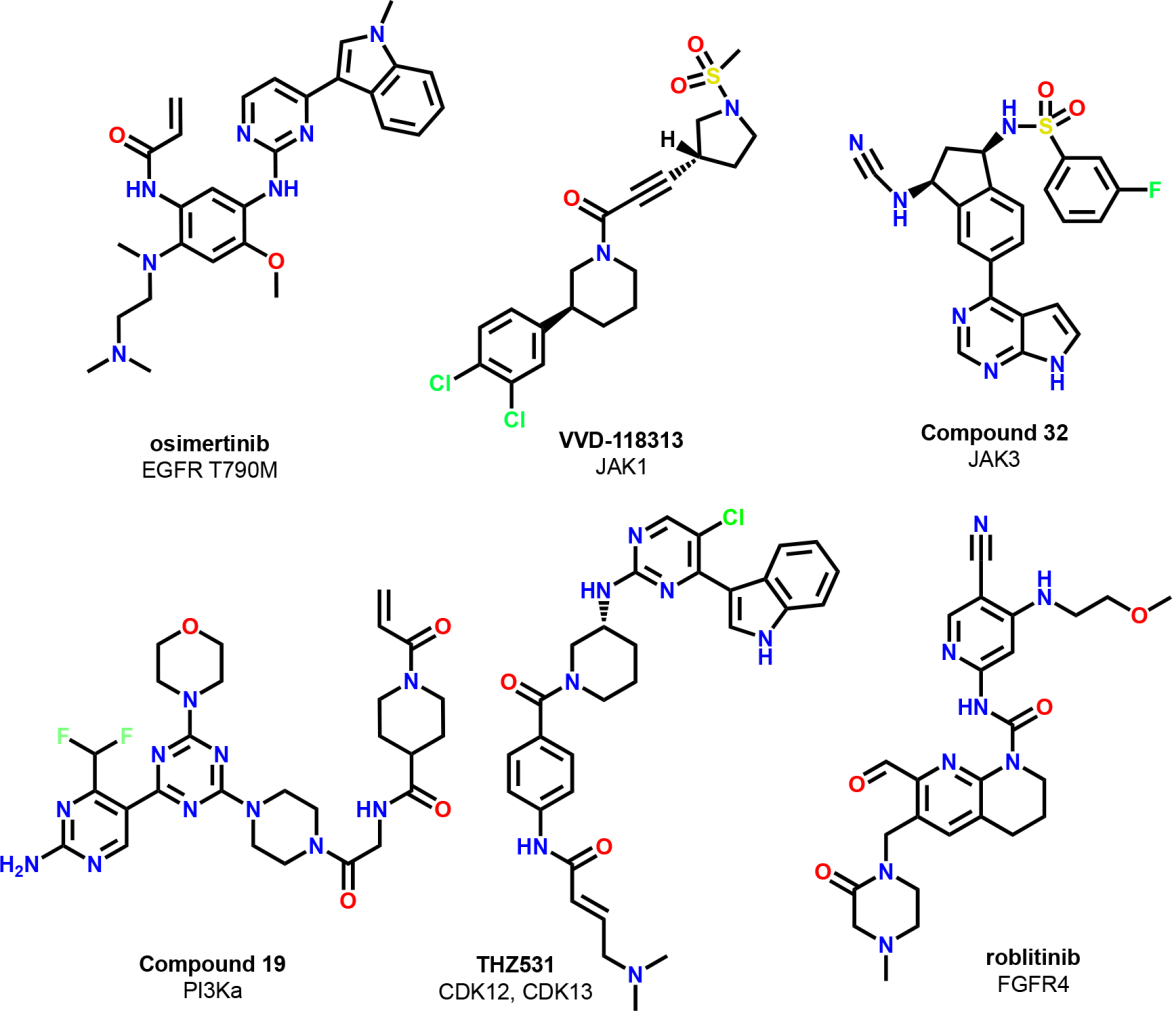
Chemical structures of high-quality covalent probes.

Generating a full validation data set for a high-quality covalent probe requires substantial efforts. However, these efforts have been well spent when such a probe can provide unique insights into the biology of its target. Examples of high-quality covalent drugs and probes are compiled in Figure 4. We consider all compounds listed as suitable, despite data gaps in some of the quality criteria outlined by us in this Perspective. However, in our opinion, the collective available data convey a satisfactory degree of confidence to ascertain that they are of high quality (e.g., indirect proof of high selectivity or position of labeling and no opposing data found in the public domain). We believe that with broader acceptance of quality criteria, as outlined in this publication, the number of published examples with a more complete data set will soon increase.

One very recent example for such a high-quality probe is the allosteric JAK1 inhibitor, VVD-118313.^41^ The compound engages JAK1 at nanomolar concentrations in cellular assays, selectively labels C817, shows only a very small number of off-targets in the relevant concentration range (Tyk2, HMOX2, SLC66A3, TOR4A), and can even be used in *in vivo* studies in animals. A highly selective covalent PI3Kα inhibitor was designed by starting from an already optimized reversible inhibitor (thereby securing high reversible binding affinity) and appending a moderately reactive warhead to reach a non-conserved distal cysteine (compound 19).^42^ Further excellent examples of high-quality covalent probes are the CDK12/13 inhibitor THZ531,^43^ and a follow-on compound, BSJ-01-175.^44^ A high-level summary of the validation data set for several of the noted examples can be found in Table 2. For all compounds, a dynamic range of SAR was demonstrated, as was biochemical selectivity to both homologous proteins and the larger gene family. While the biophysical confirmation of binding for this set is via X-ray crystallography, other valuable methods include mass spectral confirmation of exclusive modification of the PoI or a lack of affinity upon point mutation of cysteine. As can be seen in Table 2, proteome-wide selectivity data is not available in the public domain for all listed compounds, especially if the compound was already published a few years ago. The need for proteome-wide selectivity data has been critically discussed by the authors of this Perspective with broader groups of practitioners from both academic and industrial backgrounds. While we understand that the generation of such data sets requires significant time and budget investments, we are convinced that the resulting information is crucial for assessing the quality of covalent probes. Academic collaborations may help to overcome the access hurdle for such experiments.

**Table 2:** Validation Data for a Selection of Covalent Probes Which Fulfill Quality Criteria

**Compound**	**Target**	**Biochemical potency,*k***_**inact**_**/*K***_**i**_**(M**^**–1**^ **s**^**–1**^**)**	**Cellular potency**, **IC**_**50**_ **(μM)**	**Evidence of proteome-wide selectivity**	**Biophysical proof of target labeling**^n^	**Inactive control compound available?**
sotorasib^28^	KRasG12C	9,900	0.028^d^	yes	6OIM(45)	not reported
adagrasib^27^	KRasG12C	35,000	0.014^e^	yes	6USX	not reported
osimertinib^32,46^	EGFR L858R	570,000^33^	0.015^f^	not reported	6JWL, 6JXO, 6JX4, 6JXT(47)	yes^33^
VVD-118313^41^	JAK1	not reported^a^	0.032; 0.046^g^	yes	not provided	not reported
compound 32^48,49^	JAK3	190,000	0.331^h^	yes^l^	6DB4^m^	not reported
compound 19^42^	PI3Ka	414,000	0.082^i^	not reported	7R9V	yes
THZ531^44,50^	CDK12/13	not reported^b^	j	not reported	7NXJ(44)	yes, THZ513R
roblitinib^51^	FGFR4	not reported^c^	0.0043^k^	not reported	6YI8	not reported

a TEC_50_ = 0.008 μM JAK1_C817.

b IC_50_ = 0.158 μM CDK12; IC_50_ = 0.069 μM CDK13.

c IC_50_ = 0.0009 μM FGFR4.

d pERK in MIA PaCa-2.

e pERK in NCI-H358.

f pEGFR in H1975.

g IFNα-p-STAT1; IL-6-p-STAT1 in hPBMC.

h IL-15-p-STAT15 in human whole blood.

i pAKT S473 in SKOV3.

j Dose-responsive reduction of pSer2 Pol II in Jurkat cells, 50 −500 nM.

k pFGFR4 in BaF3.

l For analog, compound **6**.

m For analog, compound **34**.

n PDB code of X-ray cocrystal structure.

Most initially published covalent hits or leads for a PoI will likely not meet all the outlined criteria to sufficient an extent for use with confidence as high-quality probes. Nevertheless, we still consider such compounds valuable contributions to the scientific community and propose to categorize them as “pathfinder probes”.^5^ These compounds provide first insights into how to drug a PoI and may be used for generating X-ray crystal structures from which second-generation probes with optimized potency or selectivity can be defined. Such pathfinder probes may even be useful for deciphering aspects of target biology if they are used with caution and awareness of those aspects that may compromise the interpretation of cellular phenotypes.

One example of a covalent pathfinder probe is the KRasG12C early lead compound from the Shokat lab, “compound 12” (Figure 3). Published in 2013, this compound demonstrated a path for targeting KRasG12C and provided initial proof-of-concept in cellular assays, underscoring the therapeutic potential of targeting the GDP-bound form of KRasG12C.^52^ The approval of two KRasG12C inhibitors, sotorasib and adagrasib, for the treatment of certain types of lung cancers would have been difficult to imagine without this contribution to the scientific community.

**Figure 5:**
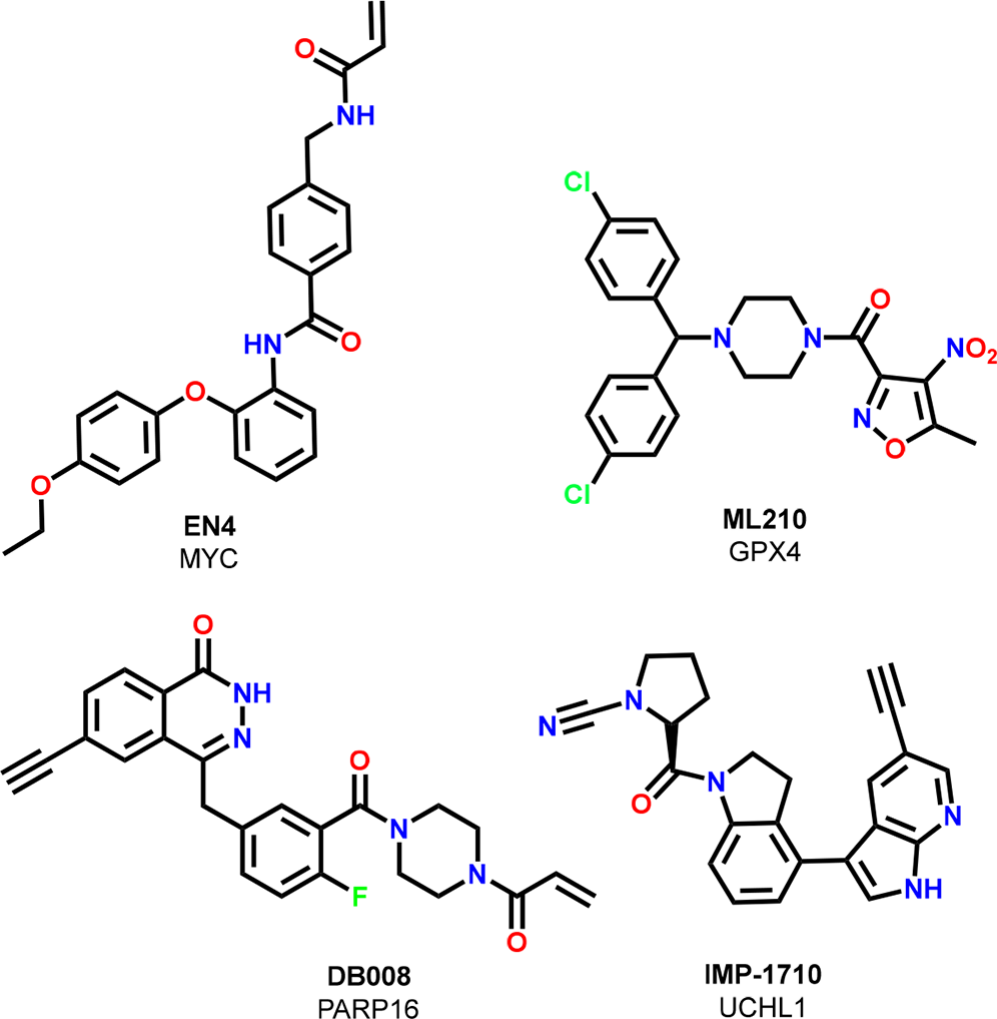
Covalent pathfinder probes.

Additional recent examples for such covalent pathfinder probes with potential impact are the GPX4 inhibitor ML210,^53^ the PARP16 inhibitor DB008,^54^ the UCHL1 inhibitor IMP-1710,^55,56^ and the cMyc binder EN4,^57^ which provide promising small-molecule entry points into engaging their respective targets (Figure 5).

With the successes in targeting cysteine residues, efforts are now underway to covalently target lysine, serine, tyrosine and arginine side chains.^58−60^ It will be important to understand whether high target selectivity can be achieved when targeting these types of side chains. A hybrid between reversibly and irreversibly acting small molecules is offered by small molecules with functionalities that allow for reversible covalent interactions with amino acid side chains. We believe that the existing framework for reversible chemical probes allows assessment of such probes, while aspects resulting from the electrophilic warhead should be assessed with the framework outlined in this Perspective in mind. Therefore, we do not see the need for a separate set of criteria for such probes.

## 3 Quality Criteria for Molecular Degrader Probes

**Figure 6:**
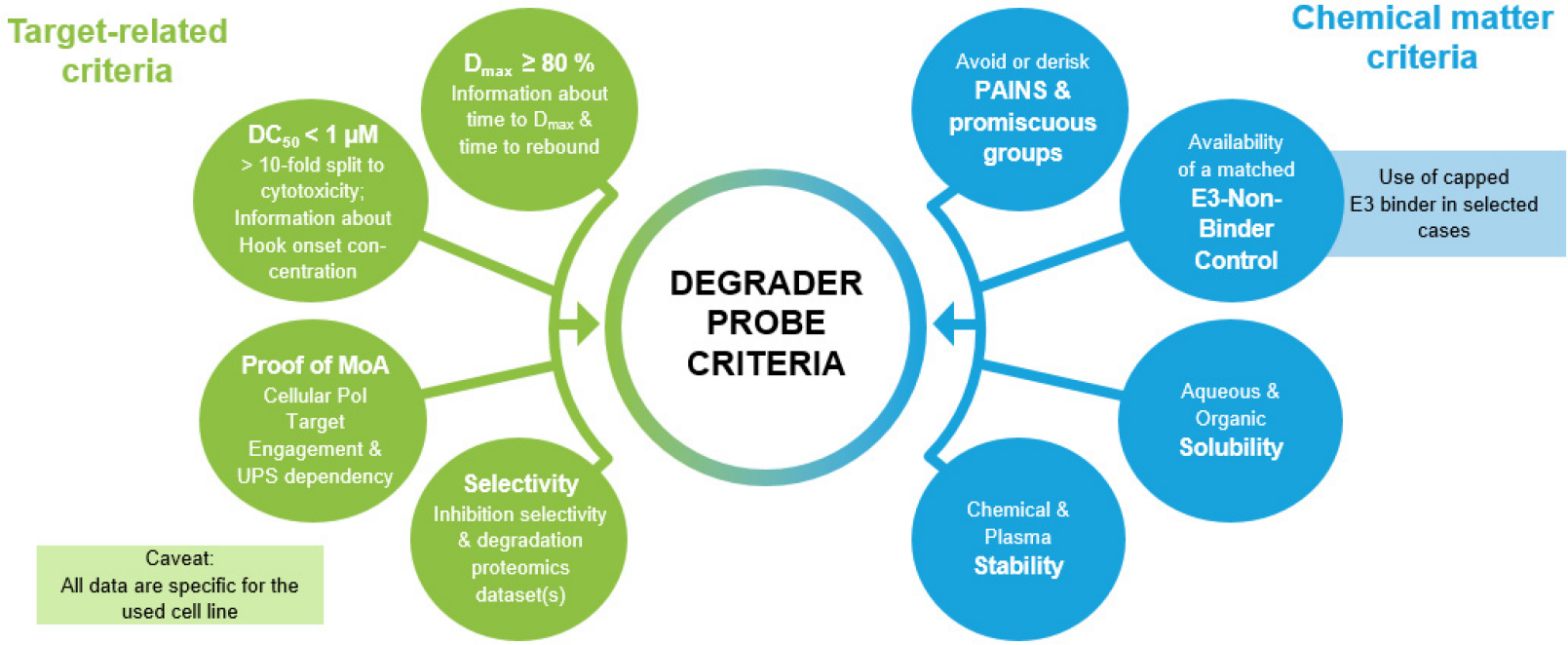
Proposed quality criteria for small-molecule degrader probes.

The following section will be dedicated to targeted protein degraders, the second modality discussed in this Perspective. We propose a set of quality criteria for small-molecule degrader probes in Figure 6. For the sake of focus, we will limit our discussion to the two most common and clinically advanced types of degraders, E3-ligase-engaging PROTACs and molecular glue degraders. However, the proposed criteria can easily be adapted to bifunctional degraders that induce proximity with effectors of the cellular protein degradation machinery other than E3 ligases.

### 3.1 Criteria for Assessing Potency of Degrader Probes

The potency of a molecular degrader is best described by the concentration necessary to achieve a 50% reduction in protein levels (DC_50_ value). In addition, the maximum achievable reduction in protein levels (within a given time frame) is an important parameter and described as the *D*_max_ value. It is important that evidence is provided that the observed degradation results from direct engagement with the PoI and not from an unknown indirect effect. Interfering with cellular fitness or the cell’s transcription/translation machinery impacts the abundance of many proteins. These general mechanisms need to be ruled out before a small molecule can be considered as a specific molecular degrader of a PoI. Implementing quality criteria will be important to avoid populating the literature with purported degraders that lead to protein depletion through off-target mechanisms rather than through a direct on-target effect.

Proving cellular target engagement at the concentration necessary to induce degradation is a good practice, and assay formats like those used for assessing cellular target engagement for reversible chemical probes can be used.^61^ PROTACs and molecular glue degraders induce a novel PPI between the PoI and an E3 ligase. Qualitative proof of the formation of a ternary complex between the degrader molecule, the PoI, and the E3 ligase provides evidence for a specific on-target mechanism. A plethora of assays have been developed to quantify PPIs both *in vitro* and in cells.^62^ While measuring ternary complex formation and cooperativity can help to drive optimization work toward more potent and more selective degraders, we do not consider generating such data to be a must-have quality criterion for degrader probes, unless other approaches fail to unequivocally rule out degradation through indirect mechanisms.

Co-incubation with a neddylation inhibitor such as MLN4924 (which inactivates most cullin-ring-ligase complexes) or a proteasome inhibitor provides proof of dependency on an active E3 ligase or the proteasome. Blocking degradation by competition with a saturating concentration of a target ligand or E3 ligase ligand can provide further evidence for on-target and on-E3 ligase activity as does the absence of degradation in cell lines devoid of the co-opted E3 ligase. An ideal scenario would be the use of an isogenic cell line pair in which one member of the pair expresses the E3 ligase, whereas the other has the E3 ligase knocked out.

We recommend the following cutoffs for the selection of high-quality molecular degrader probes: a DC_50_ below 1 μM with at least a 10-fold margin to general cytotoxicity in the same cell line. A significant consequence of the margin to cytotoxicity is that using a cell line that is highly dependent on the presence of the target of interest for survival is not a best practice for identifying target-specific degraders. As in genetic knock-down studies, it is desirable to achieve complete or near-complete target degradation, and we propose a *D*_max_ of 80% as a desirable cutoff. Even when reaching high *D*_max_ values, this may still not preclude that residual protein levels (e.g., protected against degradation by formation of tight protein complexes) are sufficient to uphold the cellular function of the protein in question.^63^

Most of the published work today uses Western blot experiments to assess protein abundance; however, caution is required especially for low-abundance proteins due to the limited dynamic range of Western blot assays. Assays using cell lines with engineered and/or overexpressed target proteins (e.g., HiBiT cell lines) can provide more granular data (including kinetic read-outs) but necessitate confirmation in physiologically more relevant non-engineered cell lines with endogenous protein and E3 ligase levels.^61^ For example, when using GFP-tagged proteins in the degradation assay, it is important to validate that the non-tagged protein is degraded as well.^64^ MS-based proteomics is emerging as a broadly used quantitative method to assess the degradation of endogenous proteins.

Sometimes underappreciated is the importance of degrader kinetics. Knowledge about the time needed to reach maximal degradation (time to *D*_max_) is crucial for designing cellular functional studies. Well-optimized molecular degraders can achieve significant effects in cells within minutes to a few hours, while less optimized ones may require up to 24 h to achieve their *D*_max_; a longer duration may also indicate that indirect mechanisms are driving the degradation of the PoI. We do not recommend specific cutoff values for time to *D*_max_ because target-specific aspects need to be considered; however, we stress that the time to *D*_max_ needs to be factored in when designing functional cellular studies. Notably, for functional cellular assays that were developed to profile conventional reversible inhibitors of an enzyme, incubation periods or read-out time points may need to be changed to assess molecular degraders of the same target protein.

As the mechanism of TPD is event- and not occupancy-driven, the effect duration is primarily determined not by continuous compound exposure but by the protein turnover rate. At *D*_max_, degradation rate and protein resynthesis rate are in equilibrium. Ideal molecular degrader tools for cellular studies achieve a *D*_max_ plateau that is stable for many hours. Information about the time when protein abundance returns to its normal level can further guide the design of cellular functional studies. One striking advantage of using molecular degraders in contrast to genetic tools to interrogate target biology is the option to observe both the onset of functional effects and the rebound to a normal state within the time frame of typical cell biology experiments.

Additional complexity when working with bifunctional molecular degraders (PROTACs) results from bell-shaped degradation–concentration curves (the so-called “hook effect”): at high degrader concentrations, induced proximity between the PoI and the E3 ligase is lost due to dominant formation of binary complexes between the degrader molecule and the desired target protein and the degrader molecule and the E3 ligase. Therefore, overdosing of bifunctional degrader probes may lead not only to undesirable off-target effects but also to decreased protein degradation. The trend to overdose probes in target validation studies is therefore of particular concern when using molecular degrader probes with small windows between DC_50_ and onset of the hook effect. In contrast to PROTACs, most molecular glue degraders show only moderate binding to one of their two intended protein binding partners and high cooperativity of the ternary complex, and therefore no bell-shaped pharmacology is to be expected.^65^

### 3.2 Criteria for Assessing Degrader Probe Selectivity

As for other types of chemical probes, selectivity needs to be carefully assessed for molecular degrader probes. Two levels of selectivity should be considered: degradation selectivity and PoI binder selectivity. It is recommended best practice to provide a whole-cell proteomics dataset for a molecular degrader probe in the same cell line used for quantification of the degrader potency. The degrader should be incubated at a concentration of 10-fold above DC_50_ and an incubation time that allows it to reach *D*_max_. All proteins being depleted more than 2-fold with a *p* value of 0.05 or better should be reported. Degradation and secondary effects such as downstream protein down-regulation can be deconvoluted in more specialized proteomic experiments, and this is especially important when relatively long incubation times are used due to slow degrader kinetics.^66,67^

Binding to proteins independent of inducing their degradation can contribute to cellular phenotypes observed with molecule degrader probes. Therefore, it is important to provide information on binder and inhibitor selectivity. For example, many kinase-targeting PROTACs are derived from kinase inhibitors. While constructing PROTACs from such kinase inhibitors adds in many cases a level of degrader selectivity, the resulting PROTACs may still be inhibitors of many more kinases than they degrade.^68^ Therefore, PROTACs derived from promiscuous small molecules are typically not suitable as molecular degrader probes for target biology studies unless modification to a PROTAC improves kinase inhibitor selectivity.

As protein inhibition and protein degradation may follow very different kinetics, comparing early vs late time points can provide first insights on the relative contributions as can the use of neddylation or proteasome inhibitors. An emerging best practice is the use of E3-non-binding control compounds to distinguish inhibitor from degrader effects. In the case of cereblon (CRBN)-type PROTACs, N-methylated IMiDs,^69^ and in the case of von Hippel–Lindau (VHL) PROTACs, the inactive diastereomer of the VHL hydroxyproline,^70^ both devoid of affinity to their respective E3 ligases, are commonly used control compounds with high value in validation studies.

In addition, the moiety engaging an E3 ligase also needs to be considered when interpreting cellular phenotypes. Co-opting an E3 ligase for TPD can modulate the physiological function of the E3 ligase.^71^ For example, cIAP1-engaging PROTACs often induce autodegradation of cIAP1, which may modulate cellular survival pathways. MDM2-engaging PROTACs can lead to accompanying stabilization of the p53 protein, resulting in an altered cellular stress response. Engaging the E3 ligase KEAP impacts the cellular levels of the tumor suppressor Nrf2. Engagement of the E3 ligase RNF114 leads to stabilization of the cell cycle modulator p21. As more E3 ligases are described for PROTAC applications, the knowledge of cellular consequences of interfering with such new E3 ligases will initially be scarce. Treatment of cells with capped E3 binders (i.e., an E3 binder with the linker part of the PROTAC attached and an assay-stable capping group instead of the target binder) can help to identify cellular effects driven not by target protein degradation but by modulation of E3 biology.

Many CRBN-engaging PROTACs make use of thalidomide- or lenalidomide-derived E3 binding moieties, which are known to induce the degradation of diverse proteins, including proteins with essential roles in cells. Such broad degradation activity will therefore complicate the interpretation of cellular read-outs, and understanding degrader selectivity (e.g., based on proteomics studies, see above) will be key to be able to draw meaningful conclusions. IMiD SAR studies have emerged and provide a basis to select CRBN-engaging molecular entities that are free of such molecular glue degradation activity.^72^ Sometimes differences in degradation kinetics (e.g., fast onset of molecular glue-derived degradation vs slower onset of PROTAC-derived degradation) can provide hints about causative links for observed cellular phenotypes.

More recently, covalent warheads have been used to co-opt E3 ligases or PoIs for TPD applications.^73^ For these warheads, the criteria discussed above for covalent chemical probes need to be considered, especially aspects about the kinetics of target labeling. Assessment of degrader selectivity will not be sufficient to guide interpretation of cellular phenotypes, as many of the first-generation covalent E3 engagers are based on highly reactive electrophiles with the potential to covalently label other proteins besides the intended E3 ligase.

For molecular glue degraders, target binding and E3 biology also need to be considered. For example, several series of cyclin K degraders have been described that are derived from CDK12/13 inhibitors.^74^ Their cellular phenotypes can be expected to be a composite of cyclin K degradation and inhibition of various CDKs, suggesting future studies to dissect the inhibitor and degrader SARs. Many thalidomide and lenalidomide derivatives induce the degradation of multiple degron-containing proteins, lead to polypharmacological effects, and are therefore unfit for use as chemical probes.

While for reversible molecular probes potency data can in most cases be transferred from one cell line to a different one, this is not that straightforward for molecular degrader probes. As mentioned, induced degradation is dependent on sufficient expression of components of the recruited E3 complex, abundance of the PoI, and cell-type-specific or cell-status-specific synthesis rates of the desired target protein. In fact, selecting E3 ligases with restricted expression profiles provides protection for tissues from the effects of degradation. For example, targeting the E3 ligase VHL enabled the identification of platelet-sparing Bcl-XL PROTACs.^75^ Therefore, before using a degrader probe in a new cell line, a study of the concentration and time dependencies of degradation of the PoI is warranted. Species differences also need to be taken into consideration, e.g., the well-documented differences for CRBN between rodent and human cells, which lead to differences of IMiD-based degraders in their ability to recruit substrate proteins.^76^ These differences are especially impactful when rodent cell lines are used to assess functional aspects of CRBN-targeting PROTACs or molecular glue degraders. While we do not discuss *in vivo* aspects in this Perspective in detail, differences between mice and human CRBN need to be kept in mind when assessing safety aspects in animals *in vivo*, and experimental set-ups have been proposed to cover safety-relevant targets in proteomics experiments.^77^

### 3.3 Chemical Matter Criteria for Degrader Probes

When selecting chemical degrader probes, it is recommended that a chemist critically assesses the chemical structure of the degrader for the presence of chemical groups that impart polypharmacology or interfere with assay read-outs (PAINs motifs).^78^ If such motifs are present, additional de-risking efforts will likely be necessary before working with such a probe unless the necessary data have already been published.

Information about solubility in standard assay buffers or recommendations for *in vivo* vehicles in general are important quality aspects when selecting probes. PROTACs are notoriously difficult to handle due to their high molecular weight and, in many cases, high lipophilicity, leading to adhesion to proteins and surfaces. While *in vitro* permeability assays are broadly used in discovery settings to predict uptake in cells and oral absorption, for PROTACs the usefulness and predictivity of such assays has been questioned, and we therefore refrain from proposing any quality criteria based on such assays.^79^

Specific care must be taken when working with IMiD-based PROTACs or molecular glue degraders. The IMiD pharmacophore is prone to base-promoted hydrolysis, and therefore, most IMiDs have limited stability at pH > 8. Racemization of the IMiD stereocenter can provide additional complexity. While for most thalidomide derivatives racemization happens rapidly in aqueous media, for some lenalidomide derivatives the stereocenter is stable and isomers can be separated. If the rest of the molecule contains additional stereocenters, diastereomeric degrader molecules can result that can have significantly different profiles. The impact of linkers on hydrolysis stability and stereochemical integrity is not always easy to predict, and experimental assessment is warranted.

In an ideal scenario, chemical degrader probes can also be used for *in vivo* studies in animals, and much progress has been made to optimize PROTACs toward oral delivery. The specific mechanism of action (MoA) of degradation resulting from induced proximity provides additional layers of complexity when interpreting *in vivo* data—like the situation for cellular studies. Most importantly, due to being event-driven, pharmacodynamic (PD) effects can be significantly disconnected from measured exposure levels, especially for target proteins with a slow resynthesis rate. PD effects may continue for many hours to days after the compound has been cleared from the relevant tissues. A detailed discussion of recommendations on how to study pharmacokinetic–pharmacodynamic aspects for molecular glue degraders is beyond the scope of this Perspective.

In theory, bell-shaped pharmacology (“hook effect”) can be expected also in *in vivo* settings, but this has been hardly ever reported. However, the impact of the target binding moiety as well as the impact of metabolites need to be considered when interpreting *in vivo* data. As in the cellular situation, target inhibition can contribute to PD modulation or even be the primary driver for observed phenotypes. Use of an E3-non-binding control compound can help to differentiate between these two contributing factors. Metabolic cleavage of the PROTAC linker between the target binder and the E3 binder may set free target inhibitors with high potency and much higher metabolic stability, which can compete off the PROTAC from the binding site of the PoI. The net result would be an inhibitor and not a degrader phenotype.

### 3.4 Examples of High-Quality Degrader Probes

**Figure 7:**
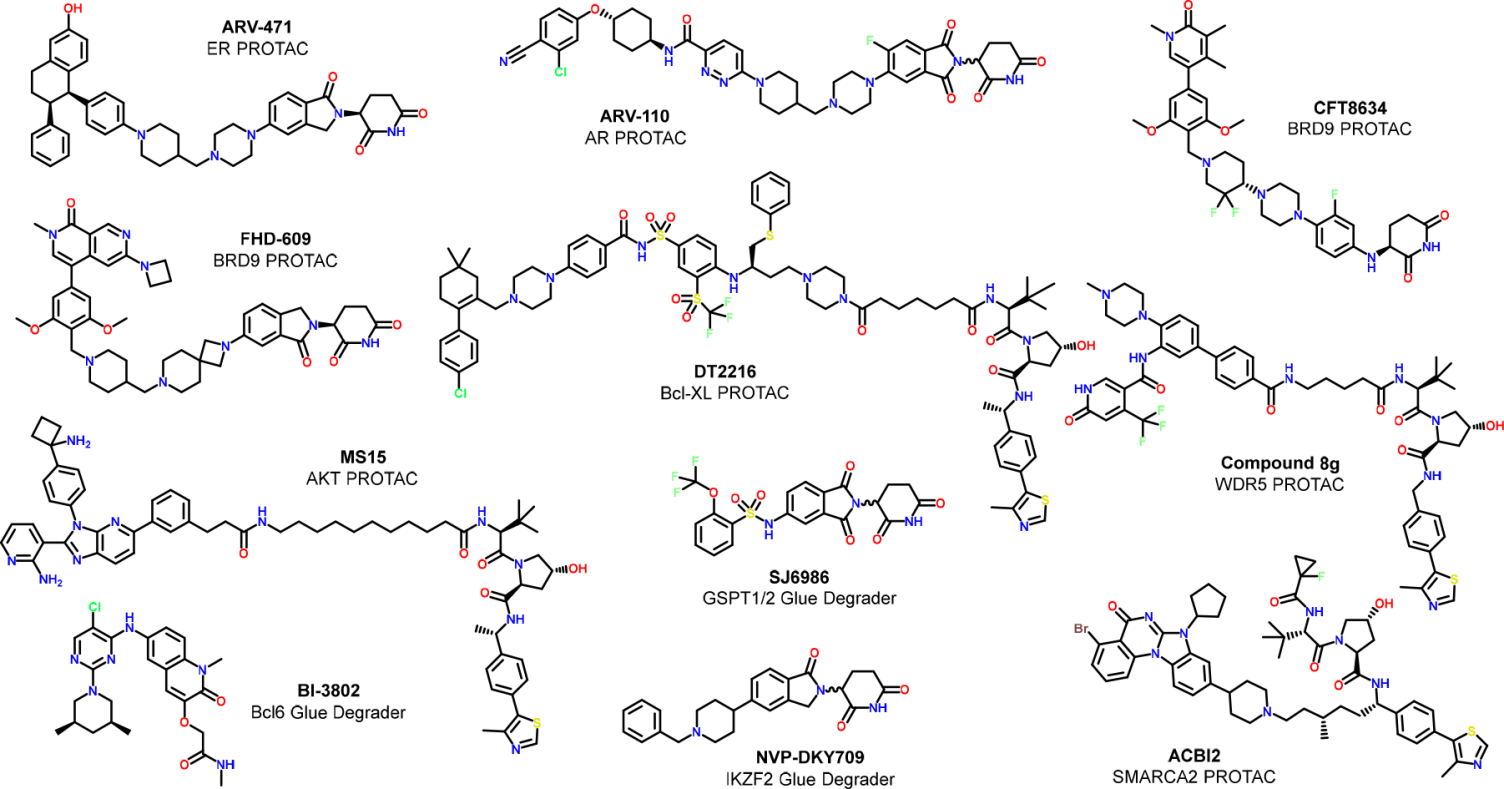
Chemical structures of high-quality degrader probes.

Though it is still early days for degrader drug discovery, there are already several fully characterized PROTACs in the public domain that could be considered as high-quality molecular degrader probes. The five clinical stage PROTACs for which chemical structures have been disclosed at the time of writing this Perspective (ARV-110, ARV-471, DDT-2216, FHD-609, and CFT-8634) all fulfill the most important criteria for high quality probes (see Figure 7 and Table 3). While degradation-inactive controls have not, to our knowledge, been published for the shown CRBN-targeting PROTACs, they would be easily accessible by methylating the CRBN-engaging IMiD motif.

**Table 3:** Validation Data for High-Quality Protein Degraders^a^

Name	Target	DC_50_ (nM)	*D*_max_ (%)	Degradation off-targets	PoI binder off-targets	Non-degrading control	Fit for p.o. use *in vivo*	Ref
ARV-110	AR	1	85			Me-imide	Y	(89)
ARV-471	ER	2	∼80			Me-imide	Y	(90)
DT2216	Bcl-X_L_	63	91		Bcl-2	DT2216NC	N	(75)
FHD-609	BRD9	<1	97		BRD7, BRD4	Me-imide	Y	(91)
CFT8634	BRD9	3	96			Me-imide	Y	(92)
ACIB2	SMARCA2	1	81	SMARCA4 (30-fold selectivity), PBRM1	SMARCA4	cis-ACBI2	Y	(81)
Cmpd 8g	WDR5	53	58			Prolinol epimer	N	(93)
MS15	AKT1	23	>80		AKT2 and AKT3	MS15N1	N	(94)
BI-3802	Bcl6	20	>80			BI-5273	N	(95)
SJ6986	GSPT1	10	90	IKZF1 (15-fold selectivity)			Y	(83)
NVP-DKY709	IKZF2	11	69	IKZF4, SALL4			Y	(84)

a DC_50_ = concentration reducing protein abundance by 50%, *D*_max_ = percentage of maximal reduction of protein abundance, Me-imide = methylated analog of CRBN-binding imide motif. PoI = protein of interest.

A noteworthy example for a high-quality research stage degrader probe is the SMARCA2 PROTAC ACBI2,^80^ which was jointly developed by the Ciulli and Boehringer Ingelheim groups and is made available to the scientific community via Boehringer Ingelheim’s OpnMe portal.^81^ ACBI2 is a VHL-based potent degrader of SMARCA2/4 with a 30-fold degradation selectivity for SMARCA2 over SMARCA4. The degrader is derived from a bromodomain inhibitor that has equal affinity for SMARCA2 and SMARCA4 and the bromodomain of PBRM1. PBRM1 is the only degrader off-target reported for ACBI2. ACBI2 achieves 20% oral bioavailability in rodents and can therefore be used *in vivo*. Cis-ACBI2 is the matched VHL-non-binder control which allows dissection of degrader from binder effects in cellular studies. The structurally distinct SMARCA2 PROTAC A947 with a similar degrader and binder selectivity profile was reported by scientists at Genentech and Arvinas.^82^

PROTACs have been published for a significant number of pharmacologically relevant targets from academic laboratories. In many cases, non-optimized PEG or alkyl linkers are used for the design of the bifunctional degrader molecule, which usually precludes oral dosing for *in vivo* studies. While non-oral routes of dosing may still allow achievement of systemic PROTAC exposure, metabolic linker lability carries the risk of generating *in vivo* phenotypes which result from multiple pharmacological active species. Many of these tool degrader molecules may be usable for cellular studies, though a critical review of the available published selectivity data (degradation selectivity, binder/inhibitor selectivity of PoI binder and E3 warhead) is necessary before use of such tools. Table 3 includes two such examples of useful degrader probes for WDR5 and AKT1.

While molecular glue degraders from the IMiD family have been tuned toward the degradation of specific target proteins, most of the structurally disclosed examples appear to not reach the necessary selectivity levels desirable for a high-quality probe (or such selectivity data have not been published). A notable exception is the GSPT1 degrader SJ6986, which shows significant selectivity against typical IMiD neosubstrates,^83^ though low expression levels of some (e.g., SALL4) in the used cell lines complicate a full selectivity assessment. Novartis has recently disclosed the profile of IKZF2 molecular glue degrader NVS-DKY709, which shows an impressive level of selectivity for IKZF2 over closely related zinc finger transcription factors IKZF1/3.^84^ In addition, DKY709 does not degrade GSPT1 but retains glue degrader activity against SALL4. There may soon be further examples of such highly selective CRBN-hijacking molecular glue degraders resulting from more extensive SAR studies to minimize the intrinsic polypharmacology of this class of molecules.

DCAF15-engaging sulfonamide degraders (e.g., indisulam) are selective degraders of the splicing factors RBM39. However, concentrations >1 μM are typically used in cellular assays to achieve high levels of RBM39 degradation and only limited selectivity data can be found in the public domain for such high concentrations.^85^ A recently published series of cyclin K degraders is derived from potent inhibitors of various CDKs (and other kinases).^86^ It will be challenging to deconvolute cyclin K degradation from kinase inhibition effects unless further SAR studies allow a separation of the degrader activity from the inhibitory potency. A highly selective Bcl6 degrader^87^ was published by scientists at Boehringer Ingelheim that makes use of a different degrader mechanism^88^ and meets the criteria for a high-quality degrader probe.

With the high interest in identifying degraders for many proteins of interest, collecting relevant information in a searchable format is important. The PROTAC database indexes over 3000 published PROTACs and includes structure, activity information, and the relevant citation. It is searchable by protein target, compound name, or compound ID.^73^ The Chemical Probes Portal (www.chemicalprobes.org) curates data associated with small-molecule probes, with a quality rating of the molecule for cellular and *in vivo* studies, based on an assessment of the published data by a team of experts.^15^ Open science depositories for degrader proteomics data and concerted efforts to make such quality checked molecular degrader probes accessible to the broader scientific community will likely be necessary to fully leverage the value of this exciting new drug modality. As for covalent probes, we expect that for many targets initially published degrader molecules will likely not meet all or sufficient quality criteria for a degrader probe but—when used with care and combined with well-designed control experiments—can also be considered as “pathfinder probes”, as they will open a route toward studying a broader section of the human proteome.

## 4 Conclusions

In this Perspective, we have proposed quality criteria for covalently acting and degrader chemical probes. For reversibly acting small-molecule probes, it took many years and continuous efforts until consensus quality criteria were broadly embraced by the relevant scientific communities. We are convinced that now is the time to initiate similar efforts to achieve a consensus about quality criteria for covalently acting and degrader probes. This Perspective is intended to jumpstart this important scientific discussion.

## Abbreviations Used

ABPP: activity-based protein profiling
CDK12: cyclin-dependent kinase 12
CDK13: cyclin-dependent kinase 13
CRBN: cereblon
*D*_max_: maximum achievable reduction in protein levels within a given time frame
DC_50_: concentration necessary to achieve 50% reduction in protein levels
GPX4: glutathione peroxidase 4
HMOX2: heme oxygenase 2
IMiD: immuno-modulatory drug
JAK1: Janus kinase 1
*k*_inact_: the rate of inactivation of the target protein
*K*_i_: affinity for the target protein
KRasG12C: Kirsten rat sarcoma proto-oncogene GTPase, mutated glycine at position 12 by cysteine
MoA: mechanism of action
MYC: v-myc avian myelocytomatosis viral oncogene homolog
PAIN: pan-assay interference
PARP16: poly(ADP-ribose) polymerase 16
PD: pharmacodynamic
PoI: protein of interest
PPI: protein–protein interaction
PROTAC: proteolysis-inducing chimera
SAR: structure–activity relationship
SLC66A3: solute carrier family 66 member 3
SMARCA2: SWI/SNF-related, matrix-associated, actin-dependent regulator of chromatin, subfamily a, member 2
SMARCA4: SWI/SNF-related, matrix-associated, actin-dependent regulator of chromatin, subfamily a, member 4
TE_50_: concentration needed to achieve target engagement for 50% of the protein
TPD: targeted protein degradation
TOR4A: torsion family 4, member A
Tyk2: tyrosine kinase 2
VHL: von Hippel–Lindau
